# MADS transcription factors cooperate: complexities of complex formation

**DOI:** 10.1093/jxb/ery099

**Published:** 2018-04-09

**Authors:** Veronique Hugouvieux, Chloe Zubieta

**Affiliations:** Laboratoire de Physiologie Cellulaire & Végétale, CEA, Univ. Grenoble Alpes, CNRS, INRA, BIG, Grenoble, France

**Keywords:** *Arabidopsis thaliana*, coiled-coil, floral quartet, flower development, keratin-like domain, MADS-box gene, MIKC-type protein, protein–protein interaction (PPI), SEPALLATA (SEP), SEPALLATA3

## Abstract

This article comments on:

**Rümpler F, Theißen G, Melzer R. 2018.** A conserved leucine zipper-like motif accounts for strong tetramerization capabilities of SEPALLATA-like MADS-domain transcription factors. Journal of Experimental Botany **69,** 1943–1954.

**MADS family transcription factors are crucial during plant reproductive development, and have evolved a complex protein–protein interaction (PPI) network. Proteins of the SEPALLATA (SEP) clade are required for tetramer formation and can act as critical ‘hubs’ in the network.** Rümpler *et al.* (2018) **have now provided quantitative measures of the contribution of individual amino acids to cooperative DNA binding, laying a foundation for predicting MADS tetramer formation based on primary sequence. It is an important step forward in understanding how cooperativity affects processes from flowering time to floral organ identity.**

Few proteins act alone, but rather form larger intricate ‘molecular machines’ to carry out diverse cellular functions. This is true in organisms ranging from relatively simple bacteria (e.g. *E. coli*) to those that are more complex like Arabidopsis (Totir *et al.*, 2012; Bigeard *et al.*, 2014; Aryal *et al.*, 2017). Transcription factors (TFs) are important examples, with the majority acting as part of larger complexes to control gene expression (Spitz and Furlong, 2012). In the plant MADS TF family, a dramatic expansion over the course of evolution resulted in the development of a large protein–protein interaction (PPI) network consisting of homodimers, heterodimers and tetramers. Thus, MADS TFs became central to virtually every aspect of plant reproductive development.

This functional diversity is directly related to the diversity of heteromeric MADS complexes that can be formed. All MADS TFs bind DNA as dimers, but some have the ability to form tetramers and so bind DNA cooperatively at two distinct sites. This property is true for the SEPALLATA (SEP) clade, and members of this clade are able to act as hubs within the MADS PPI network and drive the formation of distinct tetrameric complexes (Honma and Goto, 2001; Pelaz *et al.*, 2001; Theissen and Saedler, 2001; Ditta *et al.*, 2004; Zahn *et al.*, 2005). The composition of these complexes determines what *cis*-elements are bound and which genes are induced or repressed, orchestrating many aspects of reproduction from flowering time to floral organ identity.

The role of the *SEP* clade has been most well-studied in floral organ development. *SEP* genes are required for the formation of the floral organs, with the *sep1 sep2 sep3 sep4* quadruple mutant producing leaves in all floral whorls (Ditta *et al.*, 2004). The ABCE model simply and elegantly describes how the overlapping expression of different MADS genes, A (*APETALA1*), B (*APETALA3* and *PISTILLATA*), C (*AGAMOUS*) and E (*SEPALLATA1-4*), results in the formation of different floral organ types (Coen and Meyerowitz, 1991; Pelaz *et al.*, 2000; Honma and Goto, 2001; Theissen, 2001). Depending on MADS-gene expression patterns, different MADS proteins will be present, resulting in the formation of different dimeric and tetrameric complexes which eventually trigger distinct organogenesis programs.

## Deciphering the MADS interaction code

Like all MADS TFs capable of tetramerizing, the SEPALLATA proteins are ‘MIKC-type’, with a modular, four-domain structure: the DNA-binding MADS ‘M’ domain, a short intervening or ‘I’ domain, a coiled-coil keratin-like ‘K’ domain and a variable, largely unstructured C-terminal or ‘C’ domain (see Box 1). The dimeric M domain is highly conserved in all eukaryotes and binds a motif of ~10 bp called a CArG box which has the consensus sequence CC(A/T)_6_GG. The IKC domains are plant-specific, exhibit less sequence conservation and provide functional diversity in the plant MADS TFs. The K domain acts as a determinant of oligomerization strength and specificity, playing roles in both dimerization and tetramerization.

**Box 1. Mutagenesis studies at the K domain of SEPALLATA3**

Extensive mutagenesis studies have been performed on the K domain of SEPALLATA3 and the effect of these mutations determined by EMSAs and/or size-exclusion chromatography. Mutations in the hydrophobic tetramerization interface result in reduced cooperative binding to DNA with multiple SEP3 binding sites. Panel (A) shows a structural composite overview of a SEP3 homotetramer with each monomer coloured differently (red, yellow, green and blue). The C-terminal domain is not shown, as no homologous structures are available for modelling. The structures of the MADS domain from MEF2 (1TQE) and K domain from SEP3 (4OX0) were used to generate the composite model shown. Transparent highlights: DNA binding domains, light yellow boxes; dimerization interfaces, light purple ovals; tetramerization interface, light blue oval.

Panel (B) shows a close-up of the dimerization and tetramerization from (A) with the yellow and green monomers transparent for clarity. Labelled residues have been mutated and characterized in terms of DNA binding and/or tetramerization (Puranik *et al.*, 2014; Rümpler *et al.*, 2018). Image rendered with PyMOL (The PyMOL Molecular Graphics System, Schrödinger: pymol.org).

The table summarizes mutations and their effects on the dimerization, tetramerization and/or DNA binding cooperativity for SEP3. References: 1, Rümpler, *et al.* (2018); 2, Silva *et al.*, 2015; 3, Puranik *et al.*, 2014.

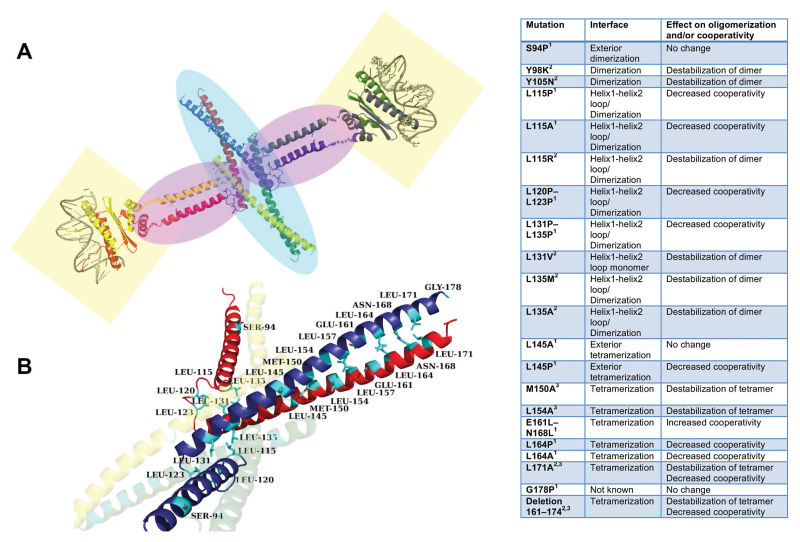

Recent studies reconstructing ancestral MADS-protein complexes place the SEPALLATAs as bridges between MADS TFs and thus as central players in the MADS PPI network. Substitutions in amino acids at certain hotspots in the K domain restrict or expand partner specificity, resulting in new interaction properties (Ruelens *et al.*, 2017). What differentiates the K domains of SEPALLATAs, allowing them to drive tetramerization with other MIKC MADS TFs, is a fundamental question in the field of plant reproductive development.

The coiled-coil domain of SEP3 responsible for the formation of tetrameric MADS complexes has recently been structurally characterized and consists of two alpha helices (Puranik *et al.*, 2014). Examination of the repeating pattern of hydrophobic residues reveals a canonical heptad repeat [*abcdefg* with the sequence HPPHPPP, respectively (H, hydrophobic; P, polar)] resulting in amphipathic helices in which one side is hydrophobic, thus providing an oligomerization surface for a partner amphipathic helix to bind, forming a coiled-coil (Crick, 1952).

In the paper by Rümpler *et al.* (2018) the authors examine the role of specific amino acids in the K domain of SEP3 (see also Box 1). The DNA-binding cooperativity of the SEP3 mutants was measured using a DNA sequence with two identical CArG boxes. These experiments revealed that even relatively conservative changes in the heptad repeat dramatically altered the ability of SEP3 to bind cooperatively to DNA, suggesting impeded tetramerization. The SEPALLATA clade conserves the heptad repeat pattern while many other MADS TFs do not to the same extent, probably preventing most MADS TFs from tetramerizing without a SEPALLATA partner. This was further demonstrated using chimeras of AP3 and SEP3 that could no longer tetramerize unless leucine residues were added at key heptad repeat positions.

## DNA binding, from *in vitro* to *in vivo*

Proper gene regulation by MADS TFs requires an intricate balance of binding events: selection of specific *cis*-elements, TF protein–protein binding and recruitment of co-factors. DNA sequence, chromatin structure and concentration of TFs and cofactors all play important roles in this process. Structural studies of the tetramerization domain of SEP3 highlight the residues likely to be important for dimer and tetramer formation (Puranik *et al.*, 2014; Silva *et al.*, 2015). The DNA sequences bound by MADS TF dimers, including SEP3, have recently been studied *in vitro* by SELEX (Smaczniak *et al.*, 2017*a* ; Smaczniak *et al.*, 2017*b* ). Cooperativity based on site spacing between *cis*-elements has also been examined through systematic variation of the number of nucleotides between DNA-binding sites (Jetha *et al.*, 2014). The extensive mutation studies by Rümpler *et al.* (2018), measuring the effects of individual amino acids on cooperativity, now add to the list of important factors contributing to MADS TF–DNA interactions. Taken together, the affinity of a given dimer for a specific target DNA sequence, the distance and orientation between putative MADS dimer-binding sites and the amino acid sequence of the coiled-coil domain of the bound MADS proteins will determine whether tetramers can form and thus whether binding is cooperative or not. Genome-wide ChIP-seq experiments of different MADS TFs can now be interpreted in the light of all these data with the goal of developing a robust predictive model for MADS TF interactions *in vivo* (Muino *et al.*, 2013).

## Effects on gene regulation and development

Detailed *in vitro* biochemical, structural and biophysical studies of MADS TFs are crucial for understanding how they function *in vivo*, and predicting the cooperative binding of MADS complexes based on the primary amino acid sequence is an important advance. The research outlined by Rümpler and colleagues lays a foundation for understanding how substitutions at key residues alter cooperative binding and change the MADS protein–protein interactome.

The wealth of *in vitro* data now available coupled with ChIP-seq experiments has given us a deeper understanding of the MADS TF–DNA binding landscape. The next step will be to examine *in vivo* the role of cooperativity in MADS TF function and gene regulation. How important cooperative binding is to gene regulation is an open question in the field and one that still needs to be answered.
